# Transcriptome Analysis Reveals Biosynthesis of Important Bioactive Constituents and Mechanism of Stem Formation of *Dendrobium huoshanense*

**DOI:** 10.1038/s41598-020-59737-2

**Published:** 2020-02-18

**Authors:** Peina Zhou, Tianzhen Pu, Chun Gui, Xiuqiao Zhang, Ling Gong

**Affiliations:** College of Pharmacy, Hubei University of Chinese Medicine, Wuhan, Hubei 430065 China

**Keywords:** Developmental biology, Genetics, Molecular biology

## Abstract

The stem of *Dendrobium huoshanense* C.Z. Tang and S.J. Cheng was widely used as a medicinal herb in health care products due to its broad pharmacological activities. However, the molecular regulation mechanism of stem development and biosynthetic pathways of important bioactive substances are still unclear in *D. huoshanense*. In this study, the bioactive compounds in leaves, stems and roots, and the identification of candidate genes involved in stem formation and biosynthesis of active compounds via transcriptome sequence were analyzed. The accumulation of total polysaccharides and flavonoids were varied significantly in different tissues. A comparative transcriptomic analysis revealed several differentially expressed genes (DEGs) involved in polysaccharides biosynthesis (103 genes), including fructose and mannose related genes (29 genes) and glycosyltransferase genes (74 genes), and flavonoids biosynthesis (15 genes). Some candidate genes that participated in photoperiod regulation (27 genes), starch and sucrose metabolism (46 genes), and hormone-induced activation of signaling pathways (38 genes) may be involved in stem formation. In sum, this study provides a foundation for investigating the molecular processes in the biosynthesis of active compounds and stem development. The transcriptome data presented here provides an important resource for the future studies of the molecular genetics and functional genomics in *D. huoshanense* and optimized control of the active compounds produced by *D. huoshanense*.

## Introduction

*Dendrobium huoshanense* C.Z. Tang and S.J. Cheng is an edible and medicinal plant, and has been utilized as a tonic in Traditional Chinese Medicine for centuries^1^. Specifically, it is used for boosting immunity, slowing aging, and possesses anti-tumor properties^2,3^. At present, increased demands and over-exploration caused reduced yield of wild resource and the damage of *D. huoshanense* habitats. Although artificial cultivation is an efficient way to protect natural resources and develop a sustainable medicinal supply, it was still faced an important issue to ensure and improve the quality and yield of *D. huoshanense* under artificial conditions. Understanding the regulation mechanism of quality and production of *D. huoshanense* has huge commercial and health value.

At present, several ingredients from *D. huoshanense* have been identified as therapeutic active components, such as polysaccharides^4,5^, flavonoids^6,7^, alkaloids^8,9^. Modern pharmacological researches have confirmed that polysaccharides can activate immune cells, modulate the immune responses in the intestines, spleen, and liver, and inhibit cell proliferation in tumors^10–13^. Alkaloids possessed the functions of anti-inflammatory, anti-diabetic effect and anticancer activity^14,15^. Flavonoids also possesses biological functions including anti-oxidant, anti-cancer, anti-aging, cardiovascular protection, and building of immunity^16–19^. The key genes associated with polysaccharides and alkaloids biosynthesis have been identified, but there are few studies centered on flavonoids biosynthesis in *D. huoshanense*. Previous work focused on identification and analysis glycosyltransferases (GTs) in polysaccharides biosynthesis^20^. Besides GTs, some metabolic pathways such as fructose and mannose metabolism may also participated in polysaccharides biosynthesis^21,22^. However, the biosynthesis mechanisms and metabolic pathways of polysaccharides have been not explored detailly in *D. huoshanense*.

In China, the stem of *Dendrobium* is a traditional edible and medicinal part^2^. However, stem growth of *D. huoshanense* requires a very harsh environment and the yield of this medicinal material was limited. Furthermore, the mechanism regulators that control the stem development of *D. huoshanense* have not been referred in the peer-reviewed publications yet. In this study, we analyzed the transcription database and measured polysaccharides and flavonoids in three tissues of *D. huoshanense*. The chemical analysis and transcriptomes provide new insights on the comprehensive characterization for biosynthesis of active ingredients in *D. huoshanense*. Furthermore, based on these transcriptomic data, we described genes and metabolism pathways associated with stem formation, which promoted understanding the regulation mechanism of stem growth and development. These results laid the solid foundation for further study to improve the quality and yield of *D. huoshanense* by expression of related genes and providing important genetic data for *Dendrobium*.

## Results

### Content of polysaccharides and flavonoids in *D. huoshanense*

**Figure 1:**
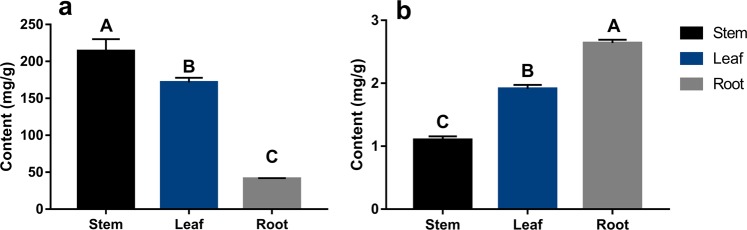
The content of total polysaccharides (**a**) and total flavonoids (**b**) in *D. huoshanense*. Different letters above bars indicate significant differences according to Fisher’s LSD test (p < 0.01).

The contents of total polysaccharides and flavonoids in different organs (leaf, stem, root) were determined in our report. In stems, the polysaccharide content was the highest (214.032 mg/g), which was 1.28 times that of found in the leaves and 5.21 times that of in the roots (Fig. 1). However, total flavonoids were mainly concentrated in leaves (2.640 mg/g) and roots (1.912 mg/g), and lowest in stems (1.102 mg/g). Statistical analysis showed that the contents of total polysaccharides and flavonoids varied significantly in different tissues.

### Illumina sequence analysis and assembly

A comprehensive overview of the *D. huoshanens*e transcriptome was carried out by constructing separate RNA-seq libraries from roots, stems, and leaves. The total number of raw reads obtained from the Illumina Hiseq 2000 platform was 476,746,678, and 444,999,698 clean reads clean reads with 66.75 Gb nucleotides remained after trimming and discarding of sequences (Supplementary Table S1). The base average error rate of was 0.01%. The average Q20 and Q30 values were 97.36 and 93.40%, respectively, and the average GC content was 47.93%. The length distribution of unigenes and transcripts in *D. huoshanense* were shown in Table 1. Among these transcript sequences, the shortest sequence was 201 bp, the longest transcript was 15,503 bp, the average length was 477 bp and the N50 was 595 bp. With an N50 length of 1,161 bp, unigenes were generated from 201 bp to 15,503 bp.

**Table 1:** Length distribution of unigenes and transcripts in *D. huoshanense*.

Nucleotide length	Transcripts	Unigenes
200–500 bp	1,163,945	177,055
500–1000 bp	206,715	180,597
1000–2000 bp	104,508	103,167
> 2000 bp	38,435	38,371
Min Length (bp)	201	201
Mean Length (bp)	477	887
Median Length (bp)	278	639
Max Length (bp)	15,503	15,503
N50 (bp)	595	1,161
N90 (bp)	228	436

**Figure 2:**
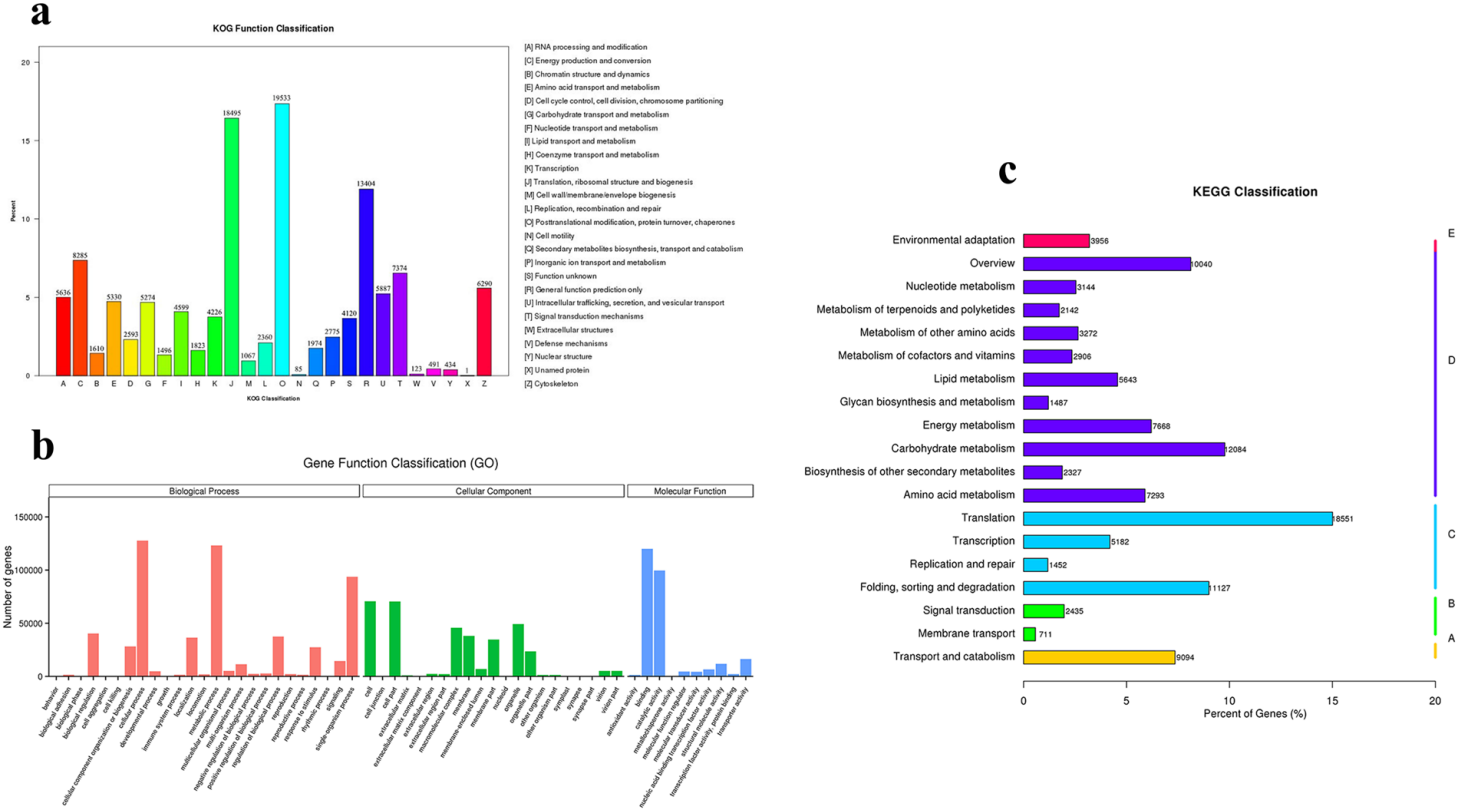
Illumina sequence analysis and assembly of *D. huoshanense*. (**a**) KOG function classification of all unigenes. (**b**) Gene ontology (GO) classification of all unigenes. (**c**) KEGG classification of all unigenes.

These assembled unigenes were then annotated using NCBI-NT, NCBI-NR, PFAM, KOG, SWISS-PROT, GO and KEGG databases, with 267,275, 140,919, 123,616, 221,707, 225,268, 228,292, and 112,603 unigenes, respectively (Table 2). In KOG, 112,603 unigenes were assigned to 26 groups (Fig. 2a). Of these, the three top terms were O (Posttranslational modification, protein turnover, chaperones, 19533), J (Translation, ribosomal structure and biogenesis, 18495) and R (General function prediction only, 13404).

**Table 2:** Unigenes annotated to the seven databases.

Component	Number of Unigenes	Percentage (%)
Annotated in NR	267,275	53.54
Annotated in NT	140,919	28.22
Annotated in KO	123,616	24.76
Annotated in SwissProt	22,1707	44.41
Annotated in PFAM	225,268	45.12
Annotated in GO	228,292	45.73
Annotated in KEGG	112,603	22.55
Annotated in all Databases	45,371	9.08
Annotated in at least one Database	332,394	66.58
Total Unigenes	499,190	100

### Analysis of functional annotation and classification by GO and KEGG

The GO analysis successfully annotated 228,292 unigenes into 56 functional terms. Among these annotated terms, 25 groups were involved in biological processes (BP), 21 groups were cellular components (CC), and 10 groups had molecular functions (MF). The majority unigenes gathered in cellular processes, metabolic processes, binding and catalytic activity terms. Metabolic processes produce several active compounds like flavonoids and polysaccharides. Key enzymes performed their catalytic function and binding in related metabolic pathways, and cellular processes may influence plant growth and development greatly. Thus, the top four GO terms may play important roles in *D. huoshanense* (Fig. 2b). For KEGG analysis, 123,616 unigenes were annotated in 131 KEGG pathways (Fig. 2c). The top three terms were “translation” (18,551 unigenes), “carbohydrate metabolism” (12,084 unigenes) and “folding, sorting and degradation” (11,127 unigenes).

### Identification of DEGs in *D. huoshanense*

**Figure 3:**
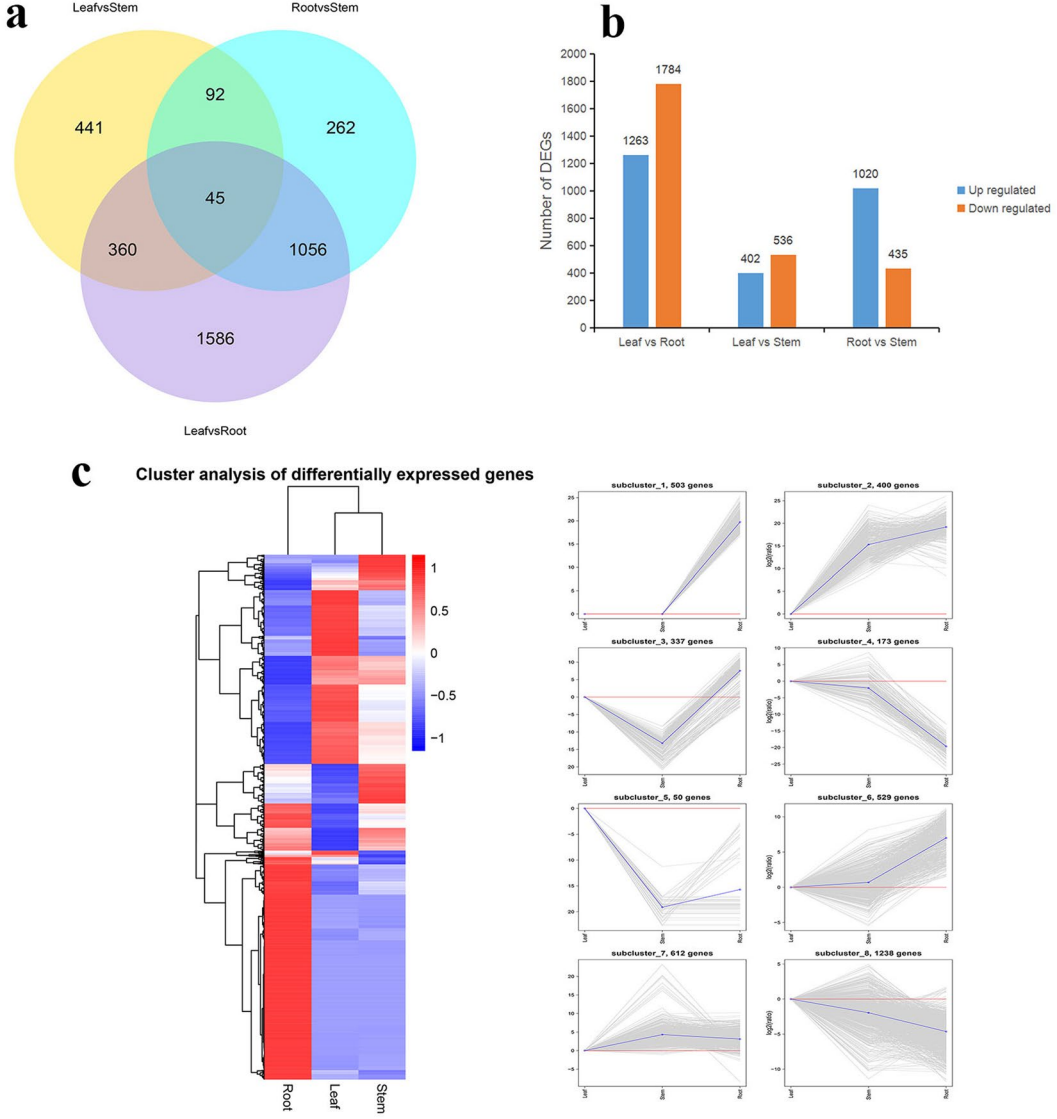
Identification of the DEGs in *D. huoshanense*. (**a**) Venn diagrams of the DEGs in S vs R, S vs F and R vs L. (**b**) The number of significantly DEGs. (**c**) Hierarchical cluster analysis of the DEGs.

Roots, stems, and leaves were compared in pairs, and respectively, 938, 1,455, and 3,047 DEGs were identified in pairs of ‘S vs. L,’ ‘S vs. R,’ and ‘R vs. L.’ As a result, 45 DEGs were expressed in all three comparisons (Fig. 3a). In detail, 137 DEGs both belonged to ‘S vs. L’ and ‘S vs. R’, 405 DEGs were in ‘S vs. L’ and ‘R vs. L,’ while DEGs in ‘S vs. R’ and ‘R vs. L’ were 1,101. In addition, 3,047 genes were expressed differentially between leaves and roots, including 1,263 up-regulated genes and 1,784 down-regulated genes. The libraries between leaves and stems showed 938 DEGs, among which 402 were up-regulated and 536 were down-regulated. Between roots and stems, 1,455 genes were differentially expressed, including 1,020 genes expressed at higher levels and 435 genes expressed at lower levels (Fig. 3b).

All the DEGs were hierarchically clustered to eight clusters using the Euclidean distance method in association with FPKM (Fig. 3c). Among eight clusters, Cluster-3 and Cluster-5 were significantly down-regulated in stems, while Cluster-7 was up-regulated. In addition, 34 up-regulated unigenes in Cluster-7 were associated with carbohydrate metabolism, RNA modification, and epigenetic regulation, which further suggested the role of these genes in coordinating global gene expression during development and the accumulation of active ingredients in *D. huoshanense* (Supplementary Table S2).

### Functional classification and functional pathways of DEGs in *D. huoshanense*

To further understand the biological function of DEGs, we analyzed their functions in pairwise comparisons with GO and KEGG pathways. In the GO analysis, there were 1,828, 650, and 1,080 DEGs annotated in ‘L vs. R,’ ‘L vs. S,’ and ‘R vs. S’ comparisons, respectively (Supplementary Fig. S1). In the three GO categories, the biological processes (BP) category was highly represented in all pairwise comparisons. The top 10 enriched GO terms in three comparisons showed that most of the DEGs belonged to multiple biological processes and molecular functions. In all comparisons, a total of eight GO terms were significantly enriched, including metabolic process, cellular process, catalytic activity, binding, organic substance metabolic process, primary metabolic process, cellular metabolic process, and single-organism process.

In the KEGG pathway analysis of DEGs, 21, 17, and 13 pathways were significantly enriched in ‘L vs. R,’ ‘L vs. S,’ and ‘R vs. S,’ respectively (P < 0.05) (Supplementary Table S3). The largest pathway that the unigenes mapped was carbon fixation in photosynthetic organisms (70 unigenes) in ‘L vs. R’. And the top enriched pathway in ‘L vs. S,’ and ‘R vs. S,’ was glyoxylate and dicarboxylate metabolism (40 unigenes) and ribosome (84 unigenes), respectively. Unigenes like metabolic genes were also enriched in the metabolic pathways of photosynthesis, phenylpropanoid biosynthesis, glycolysis/gluconeogenesis, metabolism of fructose and mannose, and pentose phosphate pathway (Supplementary Fig. S2).

### Genes related to polysaccharides biosynthesis in *D. huoshanense*

**Figure 4:**
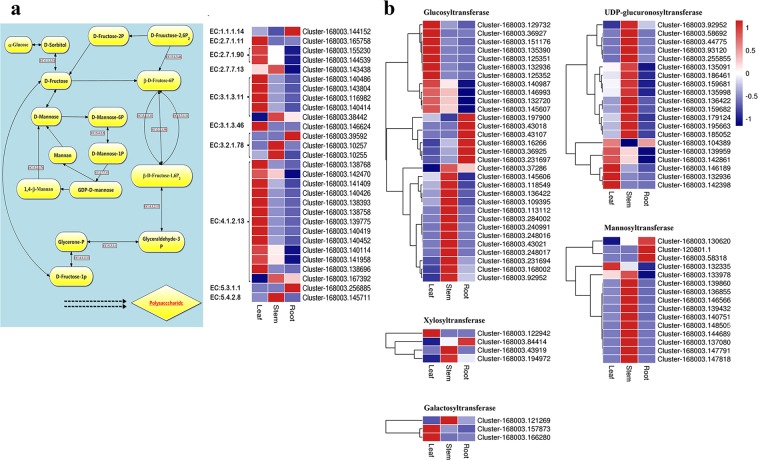
(**a**) The expression pattern of genes involved in fructose and mannose metabolism in *D. huoshanense*. The names and EC IDs of the enzymes are summarized in Supplementary Table S4. (**b**) DEGs related to glycosyltransferases in *D. huoshanense*. The gene expression values are FPKM-normalized log_2_-transformed mean transcript counts. The red and blue colors represent genes expressed at higher and lower levels, respectively.

Based on KEGG analysis, 29 unigenes were involved in fructose and mannose metabolism (Supplementary Table S4). The most numerous unigenes were identified as aldolases (EC:4.1.2.13, 13 unigenes), and the second largest number of unigenes was annotated as hexose diphosphatase (EC:3.1.3.11, 5 unigenes). Three unigenes were annotated to beta-mannanase (EC:3.2.1.78), while two unigenes were encoded as diphosphate–fructose-6-phosphate 1-phosphotransferase (EC:2.7.1.90). In addition, GDP-mannose pyrophosphorylase (EC:2.7.7.13), phosphomannose mutase (EC:5.4.2.8), fructose-2,6-bisphosphate 2-phosphatase (EC:3.1.3.46), triosephosphate isomerase (EC:5.3.1.1), phosphohexokinase (EC:2.7.1.11), and glucitol dehydrogenase (EC:1.1.1.14) were each associated with one unigene (Fig. 4a). Among them, mannose-1-phosphate guanylyltransferase (Cluster-168003.143438), fructose-bisphosphatase (Cluster-168003.38442), endo-1,4-beta-mannanase (Cluster-168003.10257, Cluster-168003.10255), fructose-bisphosphate aldolase (Cluster-168003.167392), and phosphomannomutase (Cluster-168003.145711) were predominantly expressed in the stems.

Glycosyltransferase (GT) is an important and functionally diverse family of enzymes in the biosynthesis of polysaccharides^23^. A total of 74 DEGs related to GTs have been identified, including 31 glucosyltransferases, 21 UDP-glucuronosyltransferases, 15 mannosyltransferases, 4 xylosyltransferases and 3 galactosyltransferases. The expression patterns of these genes were shown in Fig. 4b.

### Genes related to flavonoid biosynthesis in *D. huoshanense*

**Figure 5:**
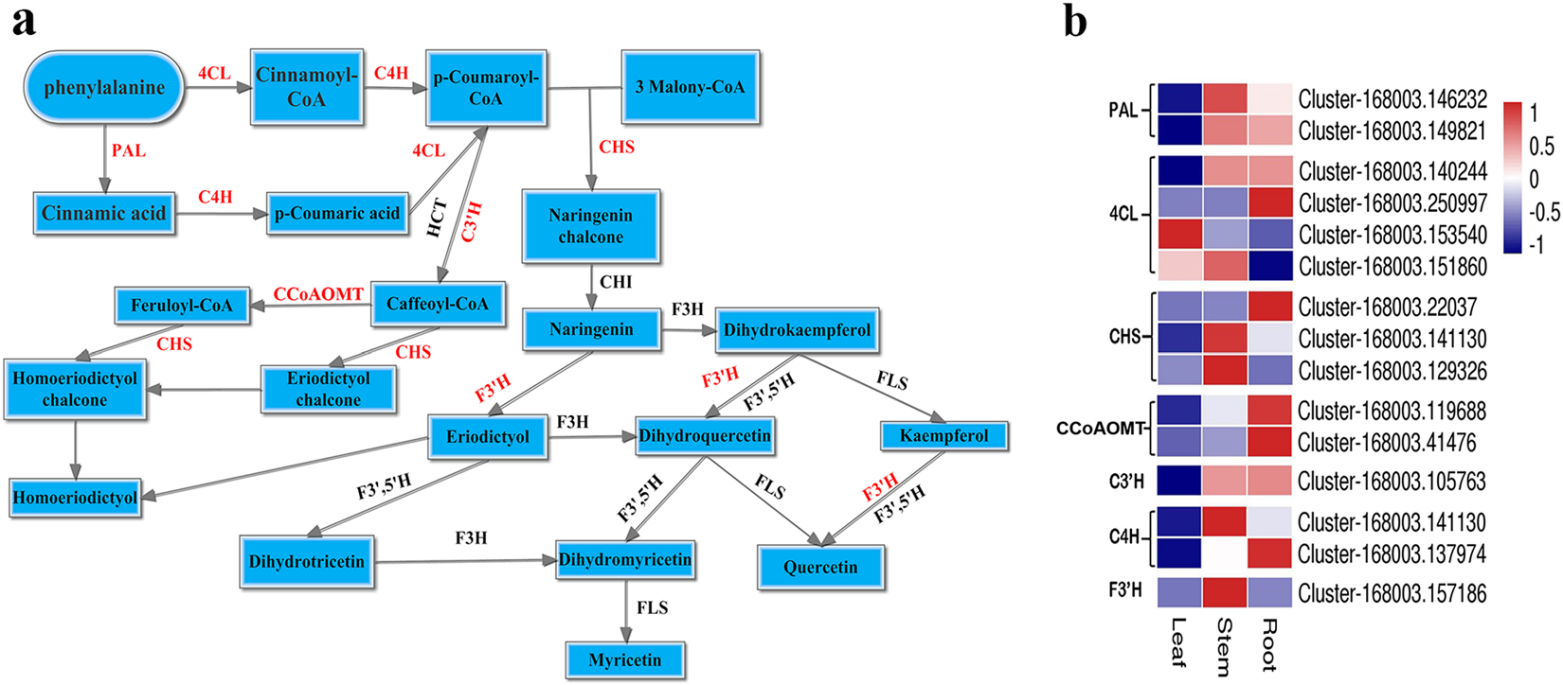
DEGs involved in the biosynthesis of flavonoids. The names and EC IDs of the enzymes are shown in Supplementary Table S5. Blue and red colors represent different levels of gene expression and the color scales reflect a log_2_-transformed mean of FPKM values.

Flavonoids are used as a new source of antioxidants that have multiple functions^24–27^. Flavonoids started with the metabolites of phenylalanine, which is catalyzed by phenylalanine ammonia-lyase (EC 4.3.1.24, PAL, 2 unigenes) to form cinnamate. After that, trans-cinnamate 4-monooxygenase (EC 1.14.13.11, C4H, 2 unigenes), and 4-coumaroyl-CoA synthase (EC 6.2.1.12, 4CL, 4 unigenes) catalyze the conversion of cinnamate to p-coumaroyl-CoA. Subsequently, chalcone, a common precursor of flavonoid, was produced from p-coumaroyl-CoA by chalcone synthase (EC 2.3.1.74, CHS, 3 unigenes). Then it can be converted to other flavonols through coumaroylquinate (coumaroylshikimate) 3′-monooxygenase (EC 1.14.13.36, C3′H, 1 unigene), flavonoid 3′-hydroxylase (EC 1.14.14.82, F3′H, 1 unigene), and trans-caffeoyl-CoA 3-O-methyltransferase (EC 2.1.1.104, CCoAOMT, 2 unigenes) (Supplementary Table S5). A majority of these unigenes were expressed in roots and stems, and a few in leaves (Fig. 5).

### Genes involved in stem development

**Figure 6:**
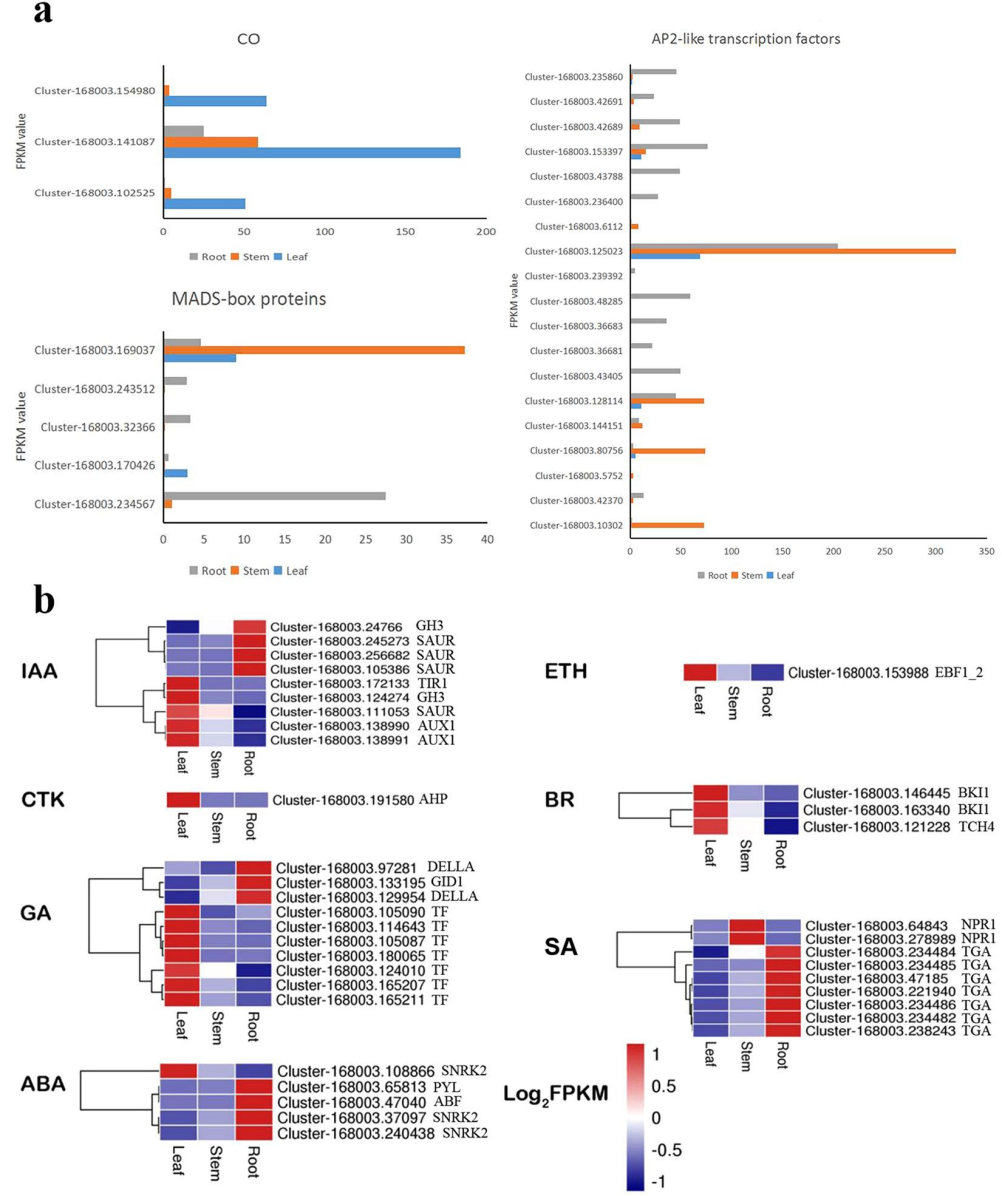
The expression levels of DEGs in stem development. (**a**) The expression levels of CO genes, five MADS-box genes and and AP2-like TFs encoding genes in *D. huoshanense*. (**b**) Heatmap of genes involved in plant hormone-induced activation of signaling pathways in the transcriptomes. The expression levels are presented as log_2_-transformed mean values ranging from −1 to 1. The red and blue color scales represent different expression levels.

Stem development is a complex physiological process that is influenced by photoperiod, metabolism of starch and sucrose, and hormone-induced activation of signaling pathways. In the photoperiod pathway, transcription factors including CONSTANS (CO, 3 unigenes), MADS-box (5 unigenes), and AP2-like (19 unigenes) were identified (Fig. 6a).

In metabolism of starch and sucrose (Supplementary Fig. S3**)**, amygdalase (BG, EC:3.2.1.21, 14 unigenes), sucrose-UDP glucosyltransferase (SUS, EC:2.4.1.13, 1 unigene), maltase-glucoamylase (MGAM, EC:3.2.1.20, 5 unigene), and β-fructofuranosidase (INV, EC:3.2.1.26, 5 unigenes) were discovered (Supplementary Table S6).

In hormone signal transduction, 38 unigenes were identified (Fig. 6b). In gibberellin (GA) signaling, key components included gibberellin receptor (GID1, 1 unigene), DELLA protein (DELLA, 2 unigenes), and phytochrome-interacting factor (TF, 7 unigenes). As for salicylic acid (SA), seven unigenes were regarded as transcription factor TGA and two unigenes were regulatory protein NPR1. Numerous genes that were regulated by abscisic acid (ABA) pathway, including PYL/PYR, SnRK2, and ABF, were differentially expressed in the samples. In indole-3-acetic acid (IAA) signaling, two unigenes, one unigene, two unigenes, and five unigenes were annotated to encode AUX1, TIR1, GH, and SAUR protein, respectively.

### Identification of TF families in *D. huoshanense*

Transcription factors (TFs) are important elements which regulate the time, location and extent of gene expression. In our study, 12,544 TFs from 81 families were detected (Supplementary Fig. S4). 2,844 of them were up-regulated in the stems, which could be grouped into 79 transcription factor families. The C2H2 (194 unigenes), the largest one, was known as their regulation on plant morphogenesis, transcriptional activation, stress and other biological processes. MYB (174 unigenes) and bHLH (123 unigenes) play critical roles in secondary metabolism in plants. Other TFs like bZIP (149 unigenes), followed by Orphans (125 unigenes), AP2-EREBP (123 unigenes), HB (123 unigenes), C3H (117 unigenes), and NAC (112 unigenes) also functioned variously.

### Validation of the expression of key genes

For expression profile analysis, 11 genes involved in fructose and mannose metabolism and 15 genes involved in flavonoid biosynthesis were selected. These data showed that the expression trends of 20 genes were roughly similar to the transcriptome (Supplementary Fig. S5a), and the correlation coefficient was 0.8176 (Supplementary Fig. S5b), which suggested that the transcriptome data were reliable.

## Discussion

*D. huoshanense* is a precious Chinese medicinal plant, however, the mechanisms responsible for active synthesis and the factors influencing stem formation are poor understand. In this study, to increase quality and yield of *D. huoshanense* by genetic improvement, we identified several putative genes involved in the biosynthesis of two active constituents, polysaccharides and flavonoids, and genes related to photoperiod, starch and sucrose metabolism, hormone signal transduction, which played an important role in stem growth.

### Identification of DEGs in polysaccharide and flavonoid biosynthesis of *D. huoshanense*

Polysaccharides were dominant substances in *D. huoshanense*, which were promising constituents for drug development due to its widely pharmacological activities^19,28^. Previous researches have showed that total polysaccharides were accumulated in the stems and accounted for 36.23% of its dry weight^29,30^. In our study, polysaccharides in *D. huoshanense* were mostly distributed in stems, which may explain why the stem is used for medicinal purposes but not roots or leaves. Analysis of the high expressed genes in stem may be useful in understanding the accumulation of polysaccharides in *D. huoshanense*. GT is a multigenic superfamily of enzymes commonly found in plants, which not only participates in glycan and glycoside biosynthesis, but also is involved in plant development, signal transduction, defense and other biological processes. They transfer sugar moieties from activated donors to specific acceptors to form glycosidic bonds which is a key downstream step for polysaccharide biosynthesis^23^. A series of key enzymes have been identified in the related reports^20,31^. In the study, we also found similar glycosyltransferases genes, including glucosyltransferases, mannosyltransferases, xylosyltransferases and galactosyltransferases. Among them, 14 glucosyltransferases unigenes, 11 mannosyltransferase unigene, 3 galactosyltransferases were highly expressed in stem, which were fourteen-fold, eleven-fold and three-fold compared to that of Yuan^20^, indicating there were more comprehensive information for polysaccharides biosynthesis studies of this species.

Mannose is one of the important components in *D. huoshanense* polysaccharide and Han *et al*. have explored six related genes in mannose biosynthetic pathway^22^. In our study, ten related genes from fructose and mannose metabolism have been explored. Among these genes, phosphomannomutase (ManB) is a key hexose metabolism enzyme that belongs to a family of phosphohexomutase. It converts mannose-6-phosphate (M-6-P) to mannose-1-phosphate (M-1-P), an important precursor of GDP-D-mannose, which is involved in the formation of glycoconjugates and protein modification^24^. The enzyme endo-1,4-beta-mannanase, which is a glycoside hydrolases, randomly cleaves the β-1,4-linkage in the mannan backbone and releases short β-1,4-mannooligosaccharides^25,26^. Mannose-1-phosphate guanylyltransferase, catalyzes D-mannose-1-phosphate to GDP-D-mannose, a precursor of mannose^27^. The three above genes were highly expressed in the stems, suggesting their essential roles in the accumulation of mannose. It deserves to carry out further studies to explore if these enzymes have special functions on polysaccharides biosynthesis.

Flavonoids are known for their high diversity, wide distribution, and multiple biological functions^32^. A previous study has shown that the leaves of *D. officinale* contained high contents of flavonoids and showed strong DPPH radical scavenging activities^30^, so that the leaves could be developed as antioxidant sources for medicinal and health food resources. Similarly, our results showed that the content of total flavonoids is concentrated in the leaves, but it is higher in the roots of *D*. *huoshanense*, suggesting that roots might also be a new source for *D. huoshanense*. Previous study has described identification and separation of chemical constituents of flavonoids (including new glycosyl flavonoids) in *D. huoshanense* by various methods^6,33^. However, limited studies have focused on the flavonoids biosynthesis in *Dendrobium* species. Here, we identified 15 unigenes encoding seven enzymes involved in flavonoid biosynthesis in *D. huoshanense*. Interestingly, these unigenes were highly expressed in roots and stems, but the content of total flavonoids was higher in roots and leaves. It would be difficult to clarify the absolute correlation between genes and the content of compounds. We speculated that the synthesis of the active compound depends on multiple genes because the biosynthesis process in plants is quite complicated and unpredictable. Apart from key genes, many proteins, like ABC transporter, SNARE, GST, CYP450, also played import roles in regulating flavonoid biosynthesis in plants^26^. Analysis of expression patterns of DEGs may be helpful to explore the accumulation of specific compounds. The roles of these genes involved in biosynthesis, regulation, and accumulation of flavonoids in plants require further investigation.

### Identification of DEGs in stem formation

The photoperiod of plants influences physiological progress and plant development, such as floral induction and tuber formation^25,34^. PHYA, PHYB, GI, FT, CO, AP2, and MADS-box genes are considered to participate in the photoperiod pathway, and the expression of these genes have an effect on the formation of the storage organ. In our study, DEGs involved in the photoperiod pathway were annotated as CO genes, MADS-box genes and AP2 genes. MADS-box genes have crucial functions for nearly all aspects of plant development, such as the formation and growth of floral organs, ovule development, and embryo development^35,36^. Among five MADS-box unigenes, they were expressed in roots, stems and leaves, and only one unigene highly expressed in stem, indicating that MADS-box genes may be not tissue specific. They may up-regulate hormone directly or indirectly to promote stem growth^37^. AP2-like transcription factors belong to the large plant-specific superfamily APETALA2 (AP2), which take part in various regulatory pathways and act in multiple stages and tissues during plant development^27^. Some members of the AP2 gene family could affect internode length, and the overexpression of an AP2-like gene in *Arabidopsis* promotes plant height^32,38^. Four unigenes expressed highly in stems and ten unigenes expressed highly in roots. It was speculated that homologous genes also differed in function and expression^39^.

Carbohydrate is important for stem development, especially starch and sucrose^40^. In our results, candidate genes in starch and sucrose metabolism pathways were selected for further analysis. Two unigenes of β-glucosidase, β-fructofuranosidase and sucrose synthase, were highly expressed in the stems. β-glucosidase hydrolyzes the terminal non-reducing residues in β-D-glucosides and oligosaccharides, with the release of glucose^41^. β-fructofuranosidase is responsible for hydrolyzing sucrose into fructose and glucose^42^. Sucrose synthase, a glycosyltransferase, can catalyze UDP-glucose to sucrose^43^. It was reported that these genes are involved in regulation of plant growth and development by providing a sustained supply of carbon^44,45^.

Plant hormones, including auxin (IAA), cytokinin (CTK), gibberellin (GA), abscisic acid (ABA), ethylene (ETH), brassinosteroid (BR), jasmonic acid (JA) and salicylic acid (SA) can regulate plant growth, development and defense. DELLA proteins are conserved inhibitors of the GA signaling pathway, which facilitates the degradation of GA via the ubiquitin proteasome^46^ and regulates all the GA-induced physiological processes in plants^47^. Two-homologous of DELLA genes were down-regulated in stems. NPR1 can positively regulate defense responses mediated by SA and induce systemic resistance; two homologous NPR1 genes were up-regulated in stems^48^. Four CTK genes, they were down-regulated in stems, suggesting postponed effects of cytokinin-related signaling pathways on senescence^49^. Moreover, several IAA, ABA, ETH, and BR related genes were detected in our study, suggesting that the developmental processes involved in stem formation in *D. huoshanense* were complicated and controlled by the possible presence of more than one gene.

### TFs related to stem formation and biosynthesis of active ingredients

TFs interacting with target genes are crucial for the growth and development of plants. They can modulate the expressions of enzyme-encoding genes and the specific accumulation of important metabolites by coordinating internal and external signals^50^. Among the 81 families of TFs detected in this research, 79 families of TFs were expressed at higher levels in the stem. C2H2, MYB, and bHLH TFs composed three of the largest TF families in plant. C2H2 is one of the main members of the zinc finger protein family and is essential for the regulation of plant growth, development, and stress resistance^51–53^. MYB factors are involved in primary and secondary metabolism, such as polysaccharide and flavonoids biosynthesis in plants, hormone signal transduction, development regulation, and response to biotic and abiotic stressors^54,55^. MYB and bHLH TFs often combined with a repeat protein WD40 to form a MYB-bHLH-WD40 complex which regulate biosynthesis of flavonoids in plants mainly^56^. Among the TFs in our results, the Orphans, AP2-EREBP, C3H, and NAC are also involved in the regulation of secondary metabolic pathways^50^. However, the underlying functions of these TFs in *D. huoshanense* need further study.

This study guides our thoughts to quantification of flavonoids and polysaccharides among three different tissues, describe gene expression profiles related to flavonoids and polysaccharides biosynthesis and mechanism of stem growth and development from the limited description of previous works. Our studies enriched the active compounds profiles of *D. huoshanense* and laid solid foundation for improving active compounds for this plant.

## Materials and Methods

### Plant materials

*Dendrobium huoshanense* plants were collected in Hubei Zongkun Dendrobium Technology Development Co., Ltd., Lei Jia Dian Zhen Dong Ming Cun, Yingshan County, Huanggang City, Hubei Province, China (30°52′43.06″N, 115°47′3.42″E). Two-year-old plants were randomly selected as the experimental materials. Fresh roots, stems, and leaves were harvested, washed, surface dried, and then immediately frozen in liquid nitrogen and kept at −80 °C for further use. All samples had three biological repeats.

### Quantification of polysaccharides and flavonoids in *D. huoshanense*

The roots, stems and leaves of *D. huoshanense* were washed and dried to constant weight at 60 °C, then grounded over a 60 mesh sieve and set aside. Total polysaccharides of *D. huoshanense* were using the phenol-sulfuric acid method^57^ and the total flavonoids were measured by NaNO_2_-Al(NO_3_)_3_-NaOH colorimetry^58^. Polysaccharides and flavonoids from *D. huoshanense* were extracted using ultrasonication. After the color reaction and complex formation, the contents were determined by ultraviolet spectrophotometry, of which the absorbance of polysaccharides was determined at 490 nm, and the flavonoids were determined at 510 nm. At the same time, the methodological investigations were conducted. D(+)-Glucose (LOT:S10S9I69833) and rutin (LOT:Y16M9S61523) were the reference substances to polysaccharides and flavonoids respectively (Shanghai Yuanye Biotechnology Co., Ltd.).

### RNA preparation, construction of cDNA libraries, and assembly

Total RNA from each tissue was extracted using the RNAprep Pure Plant Kit (Tiangen Biotech, China) following the manufacturer’s protocol. RNA integrity and quality were evaluated by denaturing RNA electrophoresis (1% agarose gel electrophoresis) and spectrophotometric analysis using a NanoDrop 2000 (Thermo Fisher Scientific, USA) with RIN number > 7.5. The extracted RNA (organs for leaves, stems, and roots) were then reverse transcribed into cDNA and sequenced. The DNA sequencing was performed using an Illumina HiSeq2000 platform (Novogene, China). After trimming the adaptors, the low-quality reads were excluded and clean reads were obtained. The Q scores, GC-content, and duplication levels of each read were determined. Only high-quality reads were subsequently analyzed. The Trinity program was used for *de novo* transcriptome assembly.

### Sequencing analysis of whole transcriptome sequencing and differentially expressed genes (DEGs)

To develop a better understanding of gene functions, unigenes were annotated using seven databases, including NCBI-NT, NCBI-NR, Pfam, KOG/COG, SWISS-PROT, GO, and KEGG. ITAK software was used to predict plant transcription factors (TF).

Transcription levels of the genes in different organs were evaluated by RSEM. We integrated the raw data for each gene into Fragments Per Kilobase Million (FPKM). Analysis of DEGs was conducted with the DESeq R package. Q value < 0.05, false discovery rate (FDR) < 0.05, and the absolute log_2_ fold-change level > 1 were assigned as differentially expressed genes^59,60^. All DEGs were divided into eight clusters and the target data were the aggregation of differential genes with the relative log_2_ values of transcript abundances. Functional enrichment analyses of DEGs were carried out with GO and KEGG databases^61–63^. GOseq R packages and KOBAS software were used to identify significantly enriched GO terms and pathways.

### Quantitative real-time PCR assays

The mRNA was isolated with the RNAprep Pure Plant Kit (Tiangen, China) and stored at −80 °C for quantitative real-time PCR (RT-qPCR). Twenty genes involved in fructose and mannose metabolism and flavonoid biosynthesis were randomly selected for RT-qPCR. All reactions were carried out in eight strip RT-PCR tubes in the Light Cycler 480 II System (Roche, USA) using the Quant One Step qRT-PCR Kit (SYBR Green) (Tiangen, Beijing, China). The PCR programs were as follows: 50 °C for 30 min, 95 °C for 2 min, and 40 rounds of 94 °C for 20 s, 55 °C for 20 s, and 68 °C for 20 s. The expression of all genes were normalized to 18S rRNA (Cluster-168003.101068) and calculated by the 2^−ΔΔCt^ method. Each gene has three biological replicates. All primers in the experience were listed in Supplementary Table S7.

### Statistical analysis

The data were collected from at least three independent repeats and expressed as the mean ± SD. Significance tests were evaluated by one-way ANOVA. The results were analyzed statistically for significance (p < 0.05) using SPSS 22.0 software (IBM Corp., NY, USA).

## Supplementary information

Supplementary Information.
