# Supplementary material for: Transcriptome Analysis Reveals Biosynthesis of Important Bioactive Constituents and Mechanism of Stem Formation of Dendrobium huoshanense
Source: Sci Rep. 2020 Feb 18;10:2857. doi: 10.1038/s41598-020-59737-2 (PMC7028924; doi:10.1038/s41598-020-59737-2)
Supplement: Supplementary file 1 — Supplementary Information. [file 41598_2020_59737_MOESM1_ESM.pdf]

SUPPLEMENTARY INFORMATION

Transcriptome Analysis Reveals Biosynthesis of Important Bioactive Constituents and Mechanism of Stem Formation of *Dendrobium huoshanense*

Peina Zhou[1], Tianzhen Pu[1], Chun Gui[1], Xiuqiao Zhang[1*], Ling Gong[1*]

[1]College of Pharmacy, Hubei University of Chinese Medicine, Wuhan, Hubei 430065, China
[*]Correspondence and requests for materials should be addressed to X.Z. (qiaoxzh2000@163.com) OR L.G. (gl1224@163.com)

Supplementary Table S1. Summary of transcriptomes from leaves, roots, and stems in *D. huoshanense.*

Sample	Raw Reads	Clean Reads	Clean Bases	Q20(%)	Q30(%)	GC Content (%)
Leaf_1	64296972	61559064	9.23G	97.63	93.91	48.43
Leaf_2	52517412	49580282	7.44G	97.37	93.41	47.03
Leaf_3	56584146	51694256	7.75G	97.35	93.37	47.87
Stem_1	45464400	40859956	6.13G	97.23	93.33	48.19
Stem_2	51914852	49196114	7.38G	97.27	93.19	48.27
Stem_3	47271714	43887466	6.58G	97.16	92.87	48.26
Root_1	53936008	50634138	7.6G	97.29	93.24	47.53
Root_2	53351144	48653916	7.3G	97.39	93.44	48.15
Root_3	51410030	48934506	7.34G	97.53	93.81	47.66
Total		444,999,698	66.75G			

Supplementary Table S2. The up-regulated unigenes in the stem in Cluster 7.

Gene ID	Description
Cluster-168003.147286	Purple acid phosphatase
Cluster-168003.279666	Putative signal transduction protein involved in RNA splicing
Cluster-168003.116304	Xaa-Pro aminopeptidase
Cluster-168003.55941	NADH:ubiquinone oxidoreductase, NDUFS2/49 kDa subunit
Cluster-168003.245794	Inorganic phosphate transporter
Cluster-168003.265741	Na+/Pi symporter
Cluster-168003.238732	Beta, beta-carotene 15,15'-dioxygenase and related enzymes
Cluster-168003.172371	Spindle pole body protein
Cluster-168003.8705	Predicted transporter (major facilitator superfamily)
Cluster-168003.237030	Aldehyde reductase
Cluster-168003.156613	Annexin
Cluster-168003.268520	Glycosyl transferase, family 8 - glycogenin
Cluster-168003.84249	Cyclin
Cluster-168003.135830	Ankyrin repeat protein

Cluster-168003.258114	Predicted E3 ubiquitin ligase
Cluster-168003.197049	Predicted hydrolase/acyltransferase (alpha/beta hydrolase superfamily)
Cluster-168003.136206	UDP-glucose 4-epimerase/UDP-sulfoquinovose synthase
Cluster-168003.139084	Multidrug resistance-associated protein/mitoxantrone resistance protein, ABC superfamily
Cluster-168003.115105	Haloacid dehalogenase-like hydrolase
Cluster-168003.50700	Aldehyde dehydrogenase
Cluster-168003.132908	Calmodulin and related proteins (EF-Hand superfamily)
Cluster-168003.181546	Long-chain acyl-CoA synthetases (AMP-forming)
Cluster-168003.150855	Ca2+-binding protein (centrin/caltractin), EF-Hand superfamily protein
Cluster-168003.149821	Phenylalanine and histidine ammonia-lyase
Cluster-168003.137974	Cytochrome oxidase subunit III and related proteins
Cluster-168003.184898	Asparaginase
Cluster-168003.198138	6-phosphogluconolactonase - like protein
Cluster-168003.134525	Long-chain acyl-CoA synthetases (AMP-forming)
Cluster-168003.156605	Annexin
Cluster-168003.145711	Phosphomannomutase
Cluster-168003.128594	Predicted glutamine synthetase
Cluster-168003.167392	Fructose-biphosphate aldolase
Cluster-168003.118361	Delta 6-fatty acid desaturase/delta-8 sphingolipid desaturase

Supplementary Table S3. The KEGG pathway analysis of DEGs in ‘L vs. R,’ ‘L vs. S,’ and ‘R vs. S.’(Table S3a-c).

Table S3a. KEGG of DEGs in ‘L vs. R’.

KEGG Term	ID	DEG number	Corrected P-Value
Photosynthesis	ko00195	54	7.32E-27
Carbon fixation in photosynthetic organisms	ko00710	70	2.64E-13
Sesquiterpenoid and triterpenoid biosynthesis	ko00909	14	2.14E-07
Photosynthesis - antenna proteins	ko00196	32	2.14E-07

Linoleic acid metabolism	ko00591	13	1.21E-06
Glyoxylate and dicarboxylate metabolism	ko00630	54	2.32E-06
Phenylpropanoid biosynthesis	ko00940	36	4.74E-06
Diterpenoid biosynthesis	ko00904	11	0.00013124
Glycine, serine and threonine metabolism	ko00260	27	0.000241826
Pentose phosphate pathway	ko00030	33	0.000241826
Stilbenoid, diarylheptanoid and gingerol biosynthesis	ko00945	10	0.002960044
Plant hormone signal transduction	ko04075	33	0.00306122
Cutin, suberine and wax biosynthesis	ko00073	8	0.00306122
Brassinosteroid biosynthesis	ko00905	7	0.00306122
Peroxisome	ko04146	35	0.003288027
Porphyrin and chlorophyll metabolism	ko00860	14	0.006399475
Cyanoamino acid metabolism	ko00460	15	0.007377965
Limonene and pinene degradation	ko00903	12	0.013732332
Fatty acid elongation	ko00062	10	0.023908409
Monoterpenoid biosynthesis	ko00902	4	0.049094196
Carotenoid biosynthesis	ko00906	9	0.049094196

Table S3b. KEGG of DEGs in ‘L vs. S’.

KEGG Term	ID	DEG number	Corrected P-Value
Glyoxylate and dicarboxylate metabolism	ko00630	40	8.98E-15
Carbon fixation in photosynthetic organisms	ko00710	32	3.18E-10
Peroxisome	ko04146	22	3.31E-06
Photosynthesis	ko00195	19	1.17E-10
Phenylpropanoid biosynthesis	ko00940	18	6.42E-06
Glycine, serine and threonine metabolism	ko00260	14	0.000112734
Cutin, suberine and wax biosynthesis	ko00073	13	1.69E-12
Fructose and mannose metabolism	ko00051	12	0.007775216

Pentose phosphate pathway	ko00030	12	0.017102031
Alanine, aspartate and glutamate metabolism	ko00250	12	0.020028162
Sesquiterpenoid and triterpenoid biosynthesis	ko00909	10	8.13E-09
Stilbenoid, diarylheptanoid and gingerol biosynthesis	ko00945	9	2.46E-06
Nitrogen metabolism	ko00910	9	0.017392756
Arginine biosynthesis	ko00220	9	0.042145656
Limonene and pinene degradation	ko00903	8	0.000905436
Carotenoid biosynthesis	ko00906	7	0.001094138
Flavonoid biosynthesis	ko00941	4	0.048239665

Table S3c. KEGG of DEGs in ‘S vs. R’.

KEGG Term	ID	DEG number	Corrected P-Value
Ribosome	ko03010	84	0.000169415
Carbon fixation in photosynthetic organisms	ko00710	23	0.001591104
Starch and sucrose metabolism	ko00500	22	0.019064137
Phenylpropanoid biosynthesis	ko00940	19	0.000106187
Photosynthesis	ko00195	18	1.87E-07
Photosynthesis - antenna proteins	ko00196	18	7.25E-06
Fatty acid elongation	ko00062	10	9.19E-05
Cyanoamino acid metabolism	ko00460	10	0.001933123
Cutin, suberine and wax biosynthesis	ko00073	8	2.44E-05
Diterpenoid biosynthesis	ko00904	7	0.000342025
Linoleic acid metabolism	ko00591	5	0.007165686
Sesquiterpenoid and triterpenoid biosynthesis	ko00909	4	0.042806672
Anthocyanin biosynthesis	ko00942	3	0.001591104

Supplementary Table S4. The unigenes involved in fructose and mannose metabolism.

Gene	Definition	EC
Cluster-168003.39592<br>Cluster-168003.10257<br>Cluster-168003.10255	beta-mannanase	EC:3.2.1.78
Cluster-168003.143438	GDP-mannose pyrophosphorylase	EC:2.7.7.13
Cluster-168003.146624	fructose-2,6-bisphosphate 2-phosphatase	EC:3.1.3.46
Cluster-168003.140486<br>Cluster-168003.143804<br>Cluster-168003.116982<br>Cluster-168003.140414<br>Cluster-168003.38442	hexose diphosphatase	EC:3.1.3.11
Cluster-168003.155230	diphosphate--fructose-6-phosphate 1-phosphotransferase	EC:2.7.1.90
Cluster-168003.144539	diphosphate--fructose-6-phosphate 1-phosphotransferase	EC:2.7.1.90
Cluster-168003.138768<br>Cluster-168003.142470<br>Cluster-168003.141409<br>Cluster-168003.140426<br>Cluster-168003.138393<br>Cluster-168003.138758<br>Cluster-168003.139775<br>Cluster-168003.140419	fructose-bisphosphate aldolase	EC:4.1.2.13

Cluster-168003.140452		
Cluster-168003.140114		
Cluster-168003.141958		
Cluster-168003.138696		
Cluster-168003.167392		
Cluster-168003.256885	triosephosphate isomerase (TIM)	EC:5.3.1.1
Cluster-168003.145711	phosphomannose mutase	EC:5.4.2.8
Cluster-168003.165758	phosphohexokinase	EC:2.7.1.11
Cluster-168003.144152	glucitol dehydrogenase	EC:1.1.1.14

Supplementary Table S5. The profile of unigenes involved in the biosynthesis of flavonoids.

Gene	Definition	EC
Cluster-168003.22037		
Cluster-168003.141130	chalcone synthase	EC:2.3.1.74
Cluster-168003.129326		
Cluster-168003.141130	trans-cinnamate-4-monooxygenase	EC:1.14.13.11
Cluster-168003.137974		
Cluster-168003.157186	flavonoid 3'-monooxygenase	EC:1.14.14.82
Cluster-168003.119688	caffeoyl-CoA O-methyltransferase	EC:2.1.1.104
Cluster-168003.41476		
Cluster-168003.105763	coumaroylquinate(coumaroylshikimate) 3'-monooxygenase	EC:1.14.13.36
Cluster-168003.146232	phenylalanine ammonia-lyase	EC:4.3.1.24
Cluster-168003.149821		
Cluster-168003.140244		
Cluster-168003.250997	4-coumarate--CoA ligase	EC:6.2.1.12
Cluster-168003.153540		
Cluster-168003.151860		

Supplementary Table S6. The details of DEGs coding key enzymes catalyzing the metabolism of starch and sucrose.

Gene	Definition
Cluster-168003.138766, Cluster-168003.146559, Cluster-168003.125699, Cluster-168003.127331<br>Cluster-168003.138856	β-fructofuranosidase
Cluster-168003.133198	maltase-glucoamylase
Cluster-168003.139302, Cluster-168003.141850<br>Cluster-168003.242107, Cluster-168003.240121<br>Cluster-168003.45174, Cluster-168003.41477, Cluster-168003.45265, Cluster-168003.236927<br>Cluster-168003.44673, Cluster-168003.43389<br>Cluster-168003.41312, Cluster-168003.241253<br>Cluster-168003.153643, Cluster-168003.131443	amygdalase
Cluster-168003.140276, Cluster-168003.146461	sucrose-phosphate synthase
Cluster-168003.150472, Cluster-168003.144462<br>Cluster-168003.162894	glucose-1-phosphate adenylyltransferase
Cluster-168003.247459, Cluster-168003.23521	glycogen phosphorylase
Cluster-168003.130535, Cluster-168003.140732<br>Cluster-168003.81710, Cluster-168003.224649<br>Cluster-168003.224651, Cluster-168003.224653<br>Cluster-168003.248068, Cluster-168003.35309	trehalose 6-phosphate phosphatase
Cluster-168003.41478, Cluster-168003.47917<br>Cluster-168003.241366	alpha,alpha-trehalase

Cluster-168003.151265	sucrose-UDP glucosyltransferase
Cluster-168003.130535, Cluster-168003.140732<br>Cluster-168003.81710, Cluster-168003.224649<br>Cluster-168003.224651, Cluster-168003.224653<br>Cluster-168003.248068	trehalose 6-phosphate synthase

Supplementary Table S7. Primers used for quantitative RT-qPCR.

Number	Gene id	Forward primer (5'-3')	Reverse primer (5'-3')
1	M1PG	GAGCCTTACACCAGATTCGACCAC	TGCTGTGATTGGAGAAGGTTGCC
2	CCOM	GCATCCTCCTCAACAAGACGTTCC	CTGCGACAACTCGATCCTCGTATC
3	C3'H	GGACTCCTTCACGACGCATTGG	ATCACAGCAGGCATGGACACAAC
4	F3'H	AATGACGGAGCTGCTAAGTGAACC	CAGACTCTTCCACGACACGATGC
5	FBA2	CCTGAAAGTCTGGGCTGAGGTGT	TGCTCGGGCGTTGCCTTATCCTT
6	FB1	CAGCACCTGTACATCAGCCATCTC	CGTAAGCATCGAAGCGAGTCTCC
7	6P1	GCAATGGCACCGGAGCTTAGG	AAGTGCGGCACATCCTCCAATAC
8	FB2	TCATCAACATCGGCGAGGCATTC	CGGTAGCGGTGGAGGAGAGC
9	PHO	TAGGTCGCCATCGCCGTGAG	TCGTCTCTGTTCACATGCGTATGG
10	LI2D	TGTGACCTTCGATTGCGCTG	GTGGCCCATTCCTACAAGGC
11	DDP	AGCTGGTCTTAATGGCTACATGGC	ATCAACAGAAGCAGGATGGACAGC
12	C4H2	CTTGGCGTCGTGGAGGTTCATG	TCCAGATCACCGAGCCAGACATC
13	C4H3	ATGCGAGATTGCGGTGATTGAGG	GGTGCTGGTTGAGAAGGCGTTA
14	PAL2	TCTCCGGTGAGTAAGGAGGC	AGGAGGTGGAGGCAACAAGA
15	4CL2	GCCAGATGGAGACTTCGGAATAGC	TTGCTGGTGCTGCTGTTGTCC
16	4CL3	GTGGTGCTCTGCGTGCTTCC	CAGCCGCCATCGTCACCTTATAC
17	4CL4	GTGGTGCTCTGCGTGCTTCC	CAGCCGCCATCGTCACCTTATAC
18	E14BM2	AGAGTTGAGTCCAGCCTCCTTAGC	TTGCAGAAGCACAGCGTCATGG
19	E14BM3	GCTTCACTCACCACGAAGTCCAG	TCGCCGCCGATCATTCAACAAG
20	FB3	CGGACGGACCTGATGACGATTAC	TGCCTTGTTGACGGCGGAAC

Supplementary Fig. S1 The GO analysis of DEGs in three comparisons.

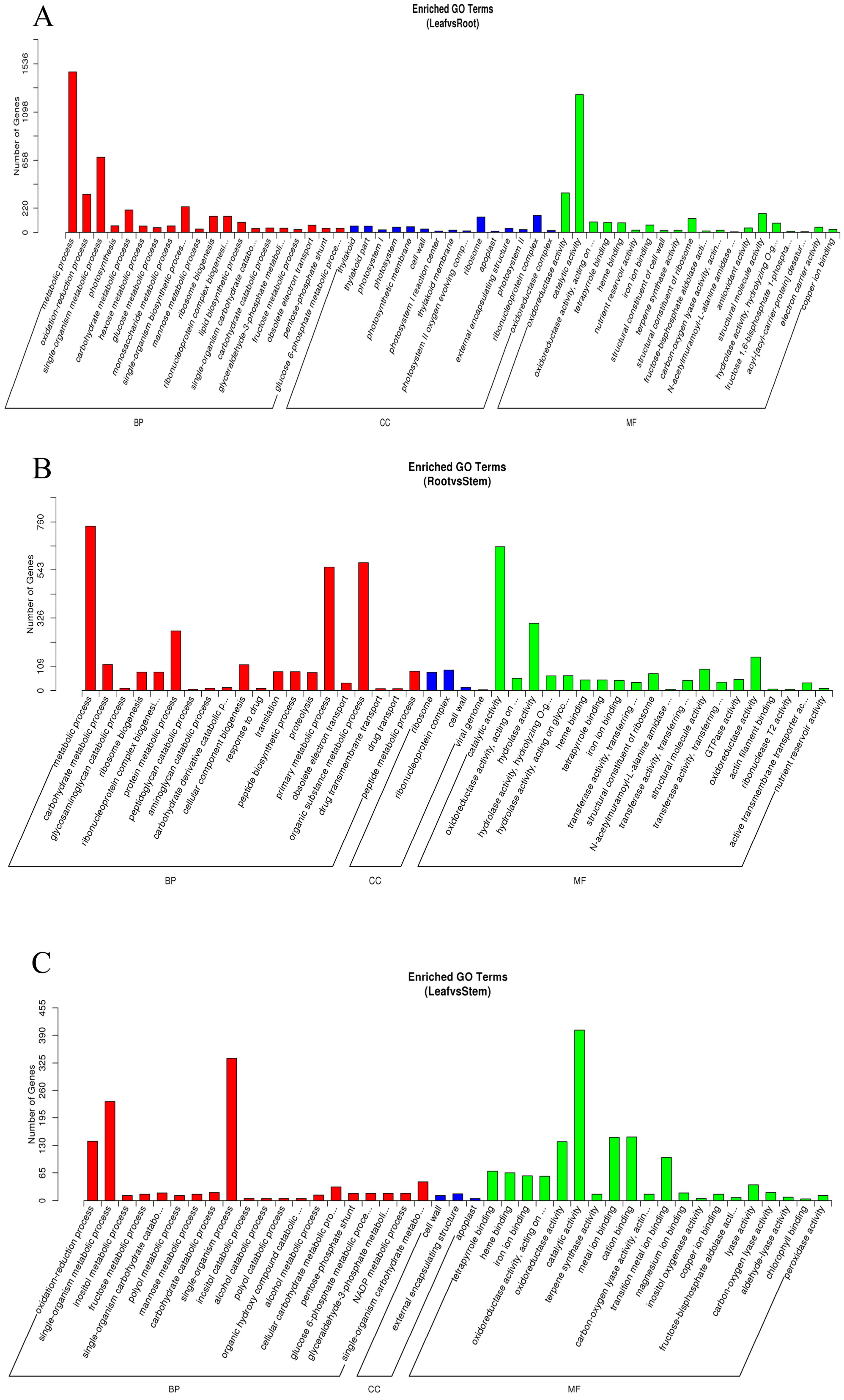

Supplementary Fig. S2 The top 20 significantly enriched KEGG pathways in DEGs in comparisons of leaves, shoots, and roots.

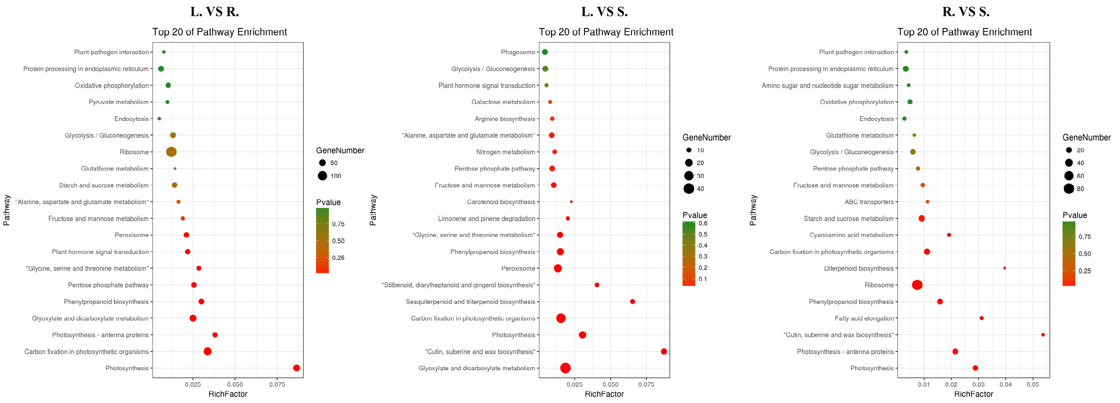

Supplementary Fig. S3 Putative starch and sucrose metabolism in DEGs; the red box indicates DEGs [63-65].

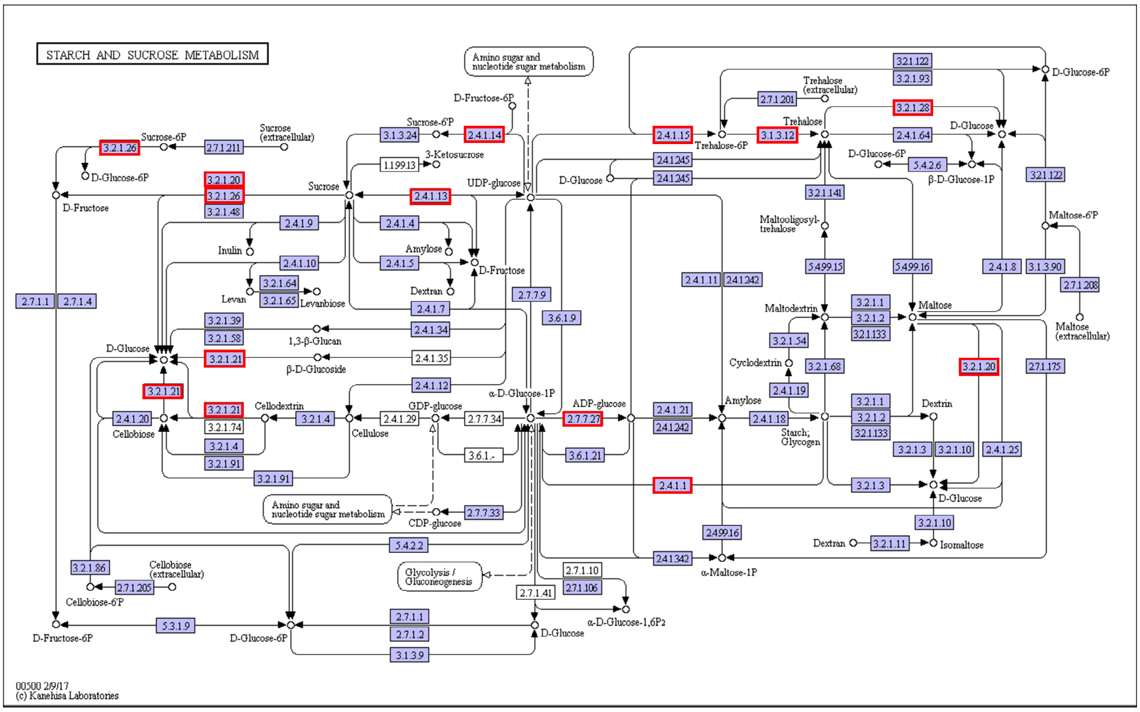

Supplementary Fig. S4 Distribution of all transcription factors.

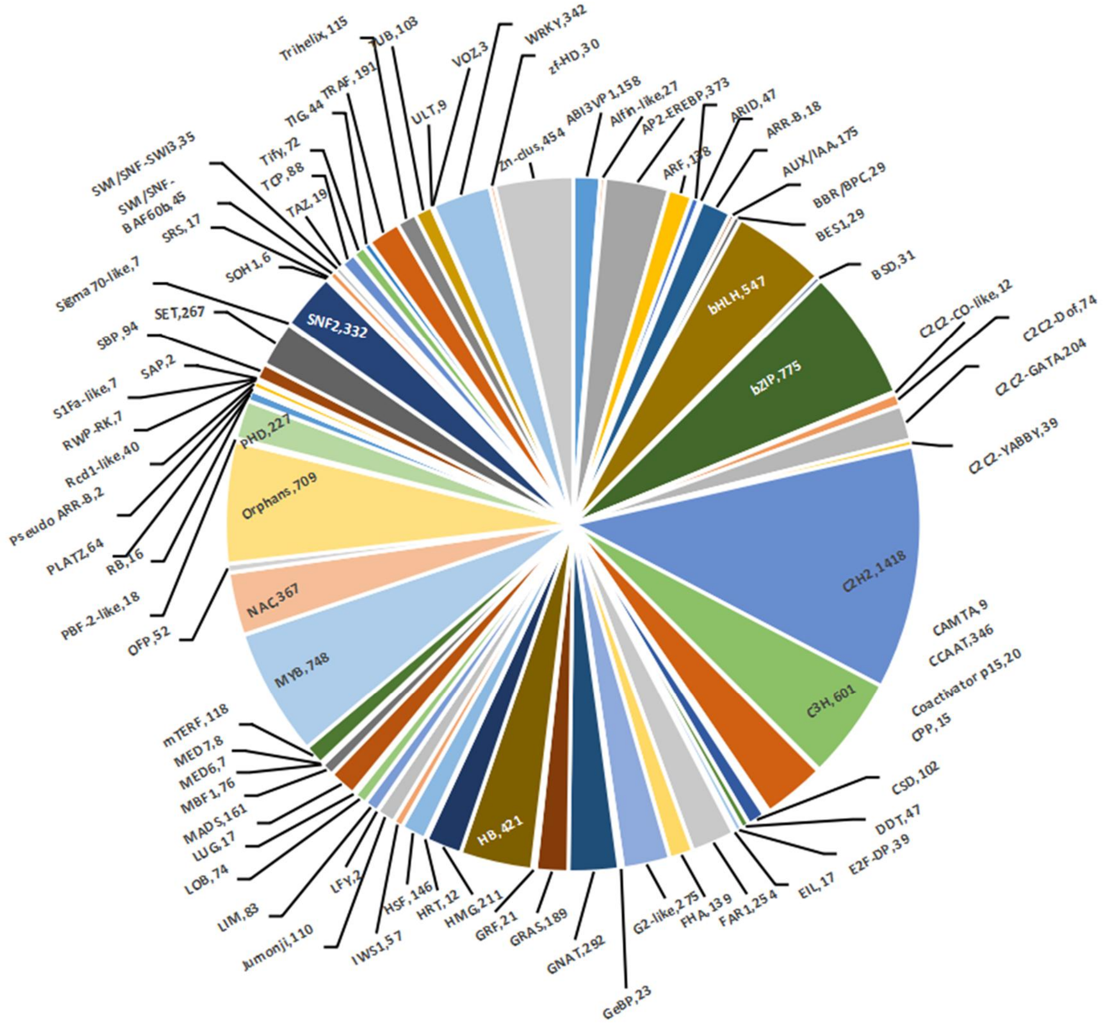

Supplementary Fig. S5 The RT-qPCR analysis of genes in fructose and mannose metabolism and flavonoid biosynthesis. (**a**) RT-qPCR was used to validate several of the differentially expressed genes identified by RNA-Seq in the three tissues of *D. huoshanense*. (**b**) The correlation between RNA-Seq and RT-qPCR.

a

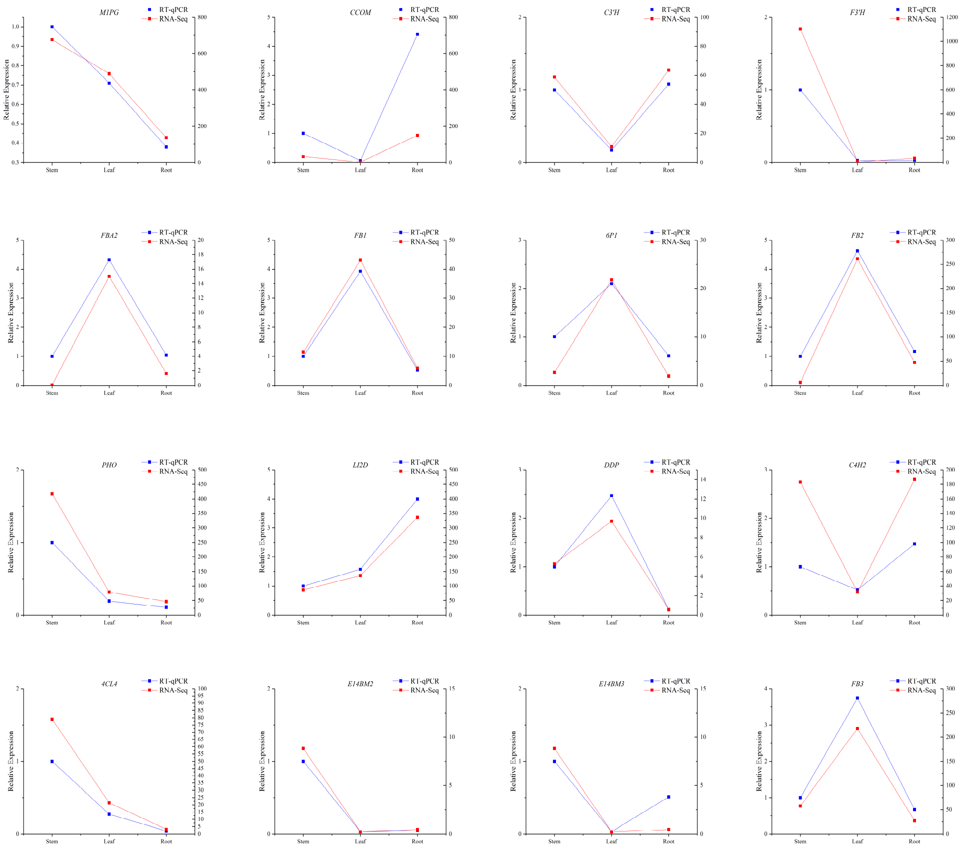
M1PG
CCOM
C3'H
F3'H
FBA2
FB1
6P1
FB2
PHO
LI2D
DDP
C4H2
4CL4
E14BM2
E14BM3
FB3
RT-qPCR
RNA-Seq
Relative Expression
Stem
Leaf
Root

b

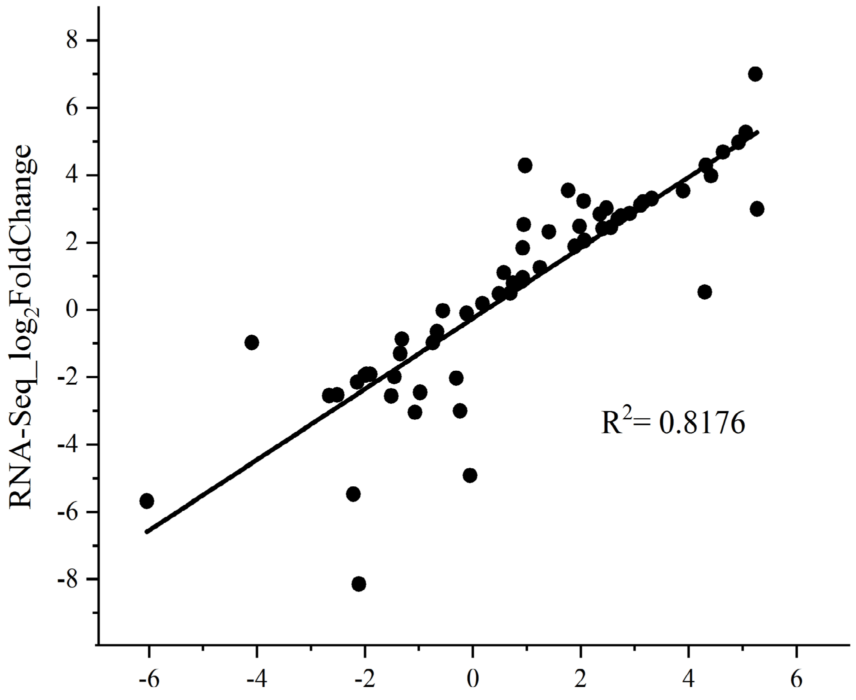
RNA-Seq_log2FoldChange
R2= 0.8176
8
6
4
2
0
-2
-4
-6
-8
-6
-4
-2
0
2
4
6
